# Computer-based cognitive tests and cerebral pathology among Japanese older adults

**DOI:** 10.1186/s12877-023-03918-x

**Published:** 2023-04-10

**Authors:** Hiroyuki Shimada, Keitaro Makino, Takashi Kato, Kengo Ito

**Affiliations:** 1 Department of Preventive Gerontology, Centre for Gerontology and Social Science, National Centre for Geriatrics and Gerontology, Aichi, Japan; 2 Department of Clinical and Experimental Neuroimaging, National Center for Geriatrics and Gerontology, Aichi, Japan

**Keywords:** Alzheimer’s disease, Beta-amyloid, Cognition, Screening, Predictor

## Abstract

**Background:**

This study aimed to identify the appropriate computer-based cognitive tests and cut-off values for estimating amyloid burden in preclinical Alzheimer’s disease drug trials.

**Methods:**

Data from 103 older individuals, who underwent ^18^F-florbetapir positron emission tomography and cognitive testing, were analyzed. Cognitive tests evaluated word list memory (immediate recognition and delayed recall), attention (Trail Making Test-part A), executive function (Trail Making Test-Part B), and processing speed (Digit Symbol Substitution Test [DSST]).

**Results:**

The Aβ burden was significantly associated with word list memory (odds ratio [OR] = 0.42, 95% confidence interval [CI], 0.19–0.91) and DSST (OR = 0.35; 95% CI, 0.14–0.85). Positive predictive value and number needed to screen at a cut-off of 1.5 SD were better for word list memory and DSST among predictive values.

**Conclusions:**

The computer-based memory and processing speed tests have the potential to reduce failure rates while screening individuals with Aβ accumulation in community settings.

## Introduction

Several failed clinical trials of beta-amyloid (Aβ)-targeting drugs suggest that intervention at very early, pre-symptomatic stages of the disease may be necessary to prevent disease progression [1]. Therefore, accurate diagnosis and timely intervention, at an early preclinical/prodromal stage of Alzheimer’s disease (AD), have been the core aims of drug development; however, the feasibility relies on identifying high-risk individuals of AD [2]. The clinical drug trials currently underway include the Anti-Amyloid Treatment in Asymptomatic Alzheimer's Disease (A4 study), including clinically normal older individuals with elevated amyloid levels on positron emission tomography (PET) scan, and the AHEAD 3–45 study, including clinically normal individuals with elevated or intermediate amyloid levels. The challenges, inherent in these prevention trials, include the difficulty in recruiting a large sample size of pre-clinical populations and the long trial durations. To select the potential subjects from older adults without cognitive decline, the A4 trial conducted amyloid PET scans on 4,486 individuals to identify 1,323 Aβ + individuals; the amyloid PET screen failure rate was 71% [3].

According to recent studies, AD pathology can be defined by plasma amyloid, tau, and neurodegeneration biomarker profiles; these profiles exhibit promising accuracy for predicting clinical progression in older adults without dementia [4]. Although brain scans, cerebrospinal fluid, or plasma biomarker profiles possess great potential in screening populations for clinical trials, cognitively normal individuals require motivation to undergo these tests. Furthermore, efforts to reduce screening failures are required to increase the efficiency of clinical trials. Computer-based cognitive tests can be performed by anyone, anytime, anywhere, and are suitable for widespread screening of populations at risk for AD. Moreover, cognitive tests are more likely to identify early abnormalities by comparing the results with age-standardized values than by using a single cut-off value to identify abnormalities. Here, we aimed to determine the suitability of computer-based cognitive tests and identify the appropriate cut-off values for age-standardized values for screening older adults with Aβ accumulation.

## Methods

### Subjects

We included 103 individuals aged ≥ 65 years (mean age, 74 years) from a sub-study of the National Centre for Geriatrics and Gerontology–Study of Geriatric Syndromes (NCGG-SGS), a national cohort study in Japan [5]. The subjects who were undergoing treatment for any substantial medical, neurological, or psychiatric disease, had clinically significant focal brain lesions on MRI, and/or scored < 21 on the Mini-Mental State Examination (MMSE) [6] were excluded. Subjects were recruited between September 2017 and December 2019, and PET and cognitive tests were performed between October 2017 and January 2020. The protocol for this study (ID: UMIN000030319) was registered in the University Hospital Medical Information Network Clinical Trials Registry website (http://www.umin.ac.jp/ctr/index.htm).

### Amyloid imaging

Aβ-PET imaging was performed with ^18^F-florbetapir. All PET scans were obtained with a PET-computed tomographic camera (Biograph 16 True Point TV, Siemens AG, Germany). Subjects underwent 3D PET imaging for 50–70 min after intravenous injection of 370 MBq ^18^F-florbetapir. The participants’ Aβ-PET dichotomization (Aβ + /Aβ −) status was visually assessed independently by two radiologists blinded to clinical or biomarker information. Consensus was obtained in case of disagreement between the two radiologists in visual reading.

### Cognitive tests

The National Centre for Geriatrics and Gerontology-Functional Assessment Tool (NCGG-FAT) [7] and the MMSE were used as cognitive tests. The NCGG-FAT has high test–retest reliability, moderate-to-high criterion-related validity [7], and predictive validity [8] among community-dwelling older persons. The NCGG-FAT has several advantages over traditional neurocognitive assessments. First, the NCGG-FAT is easily administered using a tablet PC with on-screen instructions. Therefore, it is not necessary for assessors to have in-depth knowledge of neurocognitive measures, and the individual assessor does not strongly influence the results. The simplicity and portability of the application allows assessment in community and non-clinical settings by non-specialists. Participants were able to complete the NCGG-FAT battery in approximately 20–30 min. An equivalent battery of traditional psychiatric tests would take twice as long to complete the assessment. The NCGG-FAT could be useful for cognitive screening in a population-based sample to assess the risk of cognitive decline in multidimensional functions. In addition, data collected from a large population using tablet PCs can be aggregated quickly because the data are digital rather than paper-based. The assessors of the cognitive tests were blinded to clinical information and the test results. The computer-based NCGG-FAT consists of the following domains: (1) memory (word list memory-I [immediate recognition] and word list memory-II [delayed recall]); (2) attention (an electronic tablet version of the Trail Making Test, TMT-part A); (3) executive function (an electronic tablet version of the TMT-part B); and (4) processing speed (an electronic tablet version of the Digit Symbol Substitution Test, DSST). Here, for all tests, established standardized thresholds were used to define impairment in the corresponding domain for a population-based cohort comprising 19,000 community-dwelling older persons (scores > 1.5 or > 1.0, standard deviations (SDs) below the age- and education-specific means). The MMSE score was set at an absolute of < 23, < 24, or < 26 for individuals with < 12, 12–15, or ≥ 16 years of education, respectively [9]. The MMSE is the most commonly used cognitive test around the world and was used in this study to compare the NCGG-FAT as an estimated measure of Aβ accumulation. The cognitive scores were converted to Z-scores using mean and standard deviation.

### Statistical analysis

Independent t-tests were used to compare cognitive tests between Aβ + and Aβ − subjects. Multiple logistic regression analysis was used to identify the relationships between cognitive tests, and Aβ burden was adjusted for age, sex, educational attainment, hypertension, diabetes, smoking, body mass index, living alone, and the 15-item Geriatric Depression Scale (GDS-15) [10]. We calculated accuracy, sensitivity, specificity, positive predictive value (PPV), negative predictive value (NPV), relative risk, and number needed to screen (NNS) for Aβ + status; for each test, the cut-off scores were > 1.5 or > 1.0 (SDs below the age- and education-specific means). We chose to include both a moderate level of impairment (1.5 SD) as well as a mild level of impairment (1.0 SD), which is used in mild cognitive impairment literature in an attempt to identify cognitive changes at the earliest possible point [11]. The NNS was calculated as 1/ PPV (equivalent to identifying one Aβ + individual using cognitive screening).

## Results

**Fig. 1:**
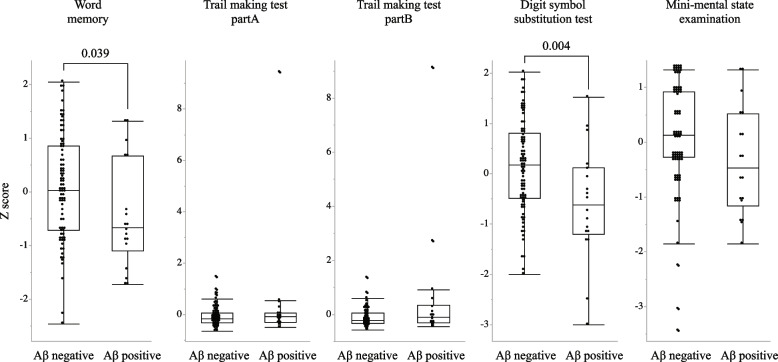
The comparison of each cognitive test between the beta-amyloid positive (Aβ +) and negative (Aβ −) groups

Of the 103 participants, 18 were Aβ + (17.5%). On comparing NCGG-FAT between the Aβ + and Aβ − groups, word list memory (*p* = 0.039) and DSST (*p* = 0.004) were significantly lower in the Aβ + group; no significant differences were observed in other cognitive tests (Fig. 1). On multiple logistic regression analysis, Aβ burden was significantly associated with word list memory (odds ratio [OR] = 0.42; 95% confidence interval [95% CI], 0.19–0.91) and DSST (OR = 0.35; 95% CI, 0.14–0.85) (Table 1).

**Table 1:** Relationships between cognitive tests and amyloid accumulation

	Hazard ratio (95% CI)	*P* value
Memory	0.42 (0.19–0.91)	0.027
TMT-A	1.28 (0.63–2.59)	0.498
TMT-B	1.81 (0.46–7.07)	0.394
SDST	0.35 (0.14–0.85)	0.020
MMSE	0.71 (0.38–1.33)	0.281

Logistic regression analysis was adjusted for age, sex, educational attainment, hypertension, diabetes, smoking, body mass index, living alone, and the 15-item Geriatric Depression Scale

*TMT-A* Trail Making Test-Part A, *TMT-B* Trail Making Test-Part B, *DSST* Digit Symbol Substitution Test, *MMSE* Mini-Mental State Examination, *SD* Standard Deviation

Table 2 shows the predicted values of each cognitive test for Aβ positivity. The accuracy, sensitivity, and specificity of each cognitive function test were 0.73–0.83, 0.06–0.39, and 0.80–0.94, respectively; relatively high predictive values were observed for word list memory and DSST. The word list memory and DSST with 1.5 SD showed PPVs of 0.45 (95% CI, 0.16–0.75) and 0.50 (95% CI, 0.22–0.78), respectively and NNS as 2.22 and 2.00, respectively; both were observed to be better predictors than the other items. At a cut-off value of 1.0 SD, although the sensitivity improved, the other predictive values decreased, and the NNS worsened for both the memory test and DSST (NNS, 3.45 and 2.63, respectively).

**Table 2:** Predictive values of cognitive function in amyloid accumulation

	Memory		TMT-A		TMT-B		DSST		MMSE
	> 1.0SD below	> 1.5SD below	> 1.0SD below	> 1.5SD below	> 1.0SD below	> 1.5SD below	> 1.0SD below	> 1.5SD below	Cutoff value
Accuracy	0.73 (0.64–0.81)	0.82 (0.74–0.89)	0.75 (0.66–0.83)	0.79 (0.71–0.87)	0.74 (0.65–0.82)	0.80 (0.72–0.87)	0.79 (0.71–0.87)	0.83 (0.75–0.90)	0.80 (0.72–0.87)
Sensitivity	0.39 (0.20–0.61)	0.28 (0.12–0.51)	0.06 (0–0.28)	0.06 (0–0.28)	0.17 (0.05–0.40)	0.17 (0.05–0.40)	0.33 (0.16–0.56)	0.33 (0.16–0.56)	0.17 (0.05–0.40)
Specificity	0.80 (0.70–0.87)	0.93 (0.85–0.97)	0.89 (0.81–0.94)	0.94 (0.87–0.98)	0.86 (0.77–0.92)	0.93 (0.85–0.97)	0.88 (0.79–0.94)	0.93 (0.85–0.97)	0.93 (0.85–0.97)
Positive predictive value	0.29 (0.11–0.47)	0.45 (0.16–0.75)	0.10 (0–0.29)	0.17 (0–0.46)	0.20 (0–0.40)	0.33 (0.03–0.64)	0.38 (0.14–0.61)	0.50 (0.22–0.78)	0.33 (0.03–0.64)
Negative predictive value	0.86 (0.78–0.94)	0.86 (0.79–0.93)	0.82 (0.74–0.90)	0.82 (0.75–0.90)	0.83 (0.75–0.91)	0.84 (0.77–0.91)	0.86 (0.79–0.93)	0.87 (0.80–0.94)	0.84 (0.77–0.91)
Relative risk	2.09 (0.94–4.67)	3.22 (1.48–7.01)	0.55 (0.12–2.55)	0.95 (0.22–4.12)	1.17 (0.42–3.28)	2.09 (0.81–5.40)	2.72 (1.24–5.98)	3.79 (1.81–7.95)	2.09 (0.81–5.40)
Number needed to screen for Aβ +	3.45	2.22	5.26	5.88	5.00	3.03	2.63	2.00	3.03

*TMT-A* Trail Making Test-Part A, *TMT-B* Trail Making Test-Part B, *DSST* Digit Symbol Substitution Test, *MMSE* Mini-Mental State Examination, *SD* Standard Deviation

## Discussion

Targeting the preclinical or prodromal stages of AD is believed to provide the best window for therapeutic intervention. ClinicalTrials.gov lists more than 450 Alzheimer's disease clinical trials requiring approximately 70,000 subjects; thus, this raises the challenge of how to efficiently identify and screen subjects [12]. Prevention trials increasingly depend on expensive brain scans or cerebrospinal fluid biomarkers to identify at-risk subjects; however, these methods have high screening failure rates because only about one-third of asymptomatic individuals may test positive [13]. According to a recent PET study, by leveraging longitudinal data in individuals classified as Aβ − at baseline, it was possible to detect early synchrony between declining memory and increasing amyloid burden [14].

Here, we compared computer-based cognitive tests to determine their suitability and identified an appropriate cut-off value for screening older adults with Aβ accumulation. The results demonstrated a significant association of Aβ burden with word list memory and DSST on multiple logistic regression analysis. The PPV of word list memory and DSST, with 1.5 SD, was 0.45 and 0.50, respectively, which was higher than those of the other items. This indicated that word list memory and DSST may be useful for screening older individuals with Aβ accumulation.

A systematic review of the diagnostic accuracy of the MMSE concluded that it may not be a suitable diagnostic tool for dementia [15] or has no advantage over shorter tests [16]. Recommendations pertaining to cognitive screening for MCI are even more uncertain [17]. Moreover, the MMSE has known limitations including its length [18], non-linearity [19], a floor effect in advanced dementia, and a ceiling effect in very mild dementia [20]. In this study, computer-based memory and processing speed tests showed better associations than the MMSE as a measure of amyloid accumulation in the brain, suggesting the greater benefit of computer-based tests in understanding brain pathology.

According to a recent study from the Trial-Ready Cohort in Preclinical/Prodromal Alzheimer’s Disease, predictive models in a web-based registry can increase the efficiency of screening in future trials for AD prevention. On A4 trial web screening test, including demographics, Cogstate brief battery, family history, and Cognitive Function Instrument, the accuracy, sensitivity, specificity, PPV, and NNS, at a standardized uptake value ratio threshold value of 1.05, were 54.9%, 60.7%, 52.8%, 31.8%, and 3.14%, respectively [21]. The NCGG-FAT showed higher PPVs in memory and DSST of 0.45 and 0.50, respectively, compared with the A4 trial web screening test findings. We concluded that the NCGG-FAT has the potential to reduce screen failure rates in Aβ + individuals to a level equal to or greater than the A4 trial web screening test. We believe that NCGG-FAT has shown excellent findings in NNS and may be useful for low-cost screening of Aβ + individuals. However, many older adults are unfamiliar with digital devices and require adequate practice to administer the test; to address this issue, the NCGG-FAT includes a practice session prior to testing.

The study had some limitations. First, the participants were not recruited randomly from the NCGG-SGS, which may have led to an underestimation of Aβ burden-prevalence; the participants were relatively healthy older individuals with the ability to access health check-up from their homes. Second, the number of individuals with Aβ accumulation in our database was limited, and a possible bias may have affected our results. Third, we did not adjust our analysis for the measurement of Apolipoprotein E, a major biomarker of Aβ accumulation, which may have contributed to the bias. Fourth, the results of this study are based on a cross-sectional study and need to be validated by prospective studies using large populations in the future.

## Data Availability

Restrictions apply to the availability of data generated or analyzed during this study to preserve participant confidentiality. The corresponding author will, on request, provide details on the restrictions and any conditions under which access to some data may be provided.

## Abbreviations

A4: Anti-Amyloid Treatment in Asymptomatic Alzheimer's Disease
Aβ: Beta-amyloid
AD: Alzheimer’s disease
DSST: Digit Symbol Substitution Test
GDS: Geriatric Depression Scale
MMSE: Mini-Mental State Examination
NCGG-FAT: National Centre for Geriatrics and Gerontology-Functional Assessment Tool
NCGG-SGS: National Centre for Geriatrics and Gerontology-Study of Geriatric Syndromes
NNS: Number needed to screen
NPV: Negative predictive value
PET: Positron emission tomography
PPV: Positive predictive value
TMT: Trail Making Test
SD: Standard deviation
